# Antimycoplasmal Activities of Compounds from *Solanum aculeastrum* and *Piliostigma thonningii* against Strains from the *Mycoplasma mycoides* Cluster

**DOI:** 10.3389/fphar.2017.00920

**Published:** 2017-12-21

**Authors:** Francisca Kama-Kama, Leonidah K. Omosa, Joseph Nganga, Naomi Maina, Georges Osanjo, Souaibou Yaouba, Muhammad Ilias, Jacob Midiwo, Jan Naessens

**Affiliations:** ^1^Department of Biochemistry, Jomo Kenyatta University of Agriculture and Technology, Nairobi, Kenya; ^2^Biosciences Eastern and Central Africa, International Livestock Research Institute, Nairobi, Kenya; ^3^Department of Chemistry, University of Nairobi, Nairobi, Kenya; ^4^Department of Pharmacology and Pharmacognosy, School of Pharmacy, University of Nairobi, Nairobi, Kenya; ^5^National Center for Natural Products Research, Research Institute of Pharmaceutical Sciences, School of Pharmacy, University of Mississippi, Oxford, MS, United States

**Keywords:** antimycoplasmal activities, *Solanum aculeastrum*, *Piliostigma thonningii*, *Mycoplasma mycoides*, glycosidic steroidal alkaloid

## Abstract

Infections caused by *Mycoplasma* species belonging to the *‘mycoides* cluster’ negatively affect the agricultural sector through losses in livestock productivity. These *Mycoplasma* strains are resistant to many conventional antibiotics due to the total lack of cell wall. Therefore, there is an urgent need to develop new antimicrobial agents from alternative sources such as medicinal plants to curb the resistance threat. Recent studies on extracts from *Solanum aculeastrum* and *Piliostigma thonningii* revealed interesting antimycoplasmal activities hence the motivation to investigate the antimycoplasmal activities of constituent compounds. The CH_2_Cl_2_/MeOH extracts from the berries of *S. aculeastrum* yielded a new β-sitosterol derivative (**1**) along with six known ones including; lupeol (**2**), two long-chain fatty alcohols namely undecyl alcohol (**3**) and lauryl alcohol (**4**); two long-chain fatty acids namely; myristic acid (**5**) and nervonic acid (**6**) as well as a glycosidic steroidal alkaloid; (25R)-3β-*O*-α-L-rhamnopyranosyl-(1→2)-*O*-[α-L-rhamnopyranosyl-(1→4)]-β-D-glucopyranosyloxy-22α-N-spirosol-5-ene (**7**) from the MeOH extracts. A new furan diglycoside, (2,5-D-diglucopyranosyloxy-furan) (**8**) was also characterized from the CH_2_Cl_2_/MeOH extract of stem bark of *P. thonningii*. The structures of the compounds were determined on the basis of spectroscopic evidence and comparison with literature data. Compounds **1, 3, 4, 7**, and **8** isolated in sufficient yields were tested against the growth of two *Mycoplasma mycoides* subsp. *mycoides* (*Mmm*), two *M. mycoides. capri* (*Mmc*), and one *M. capricolum capricolum* (*Mcc*) using broth dilution methods, while the minimum inhibitory concentration (MIC) was determined by serial dilution. The inhibition of *Mycoplasma in vitro* growth was determined by the use of both flow cytometry (FCM) and color change units (CCU) methods. Compounds **4** and **7** showed moderate activity against the growth of *Mmm* and *Mmc* but were inactive against the growth of *Mcc*. The lowest MIC value was 50 μg/ml for compound **7** against *Mmm*. The rest of the compounds showed minimal or no activity against the strains of *Mycoplasma mycoides* tested. This is the first report on the use of combined FCM and CCU to determine inhibition of *in vitro* growth of *Mycoplasma mycoides*. The activity of these compounds against other bacterial strains should be tested and their safety profiles determined.

## Introduction

*Mycoplasma*s are distinguished phenotypically from other bacteria by their small size and total lack of a cell wall (Weisburg et al., 1989). Infections caused by these *Mycoplasma* constitute a threat to both humans and animals (Arjoon et al., 2012). Many species belonging to these classes are pathogenic and of great economic concern in livestock production (Tambi et al., 2006). In particular, the so-called “*Mycoplasma mycoides* cluster” consists of ruminant pathogens (Fischer et al., 2012) and they negatively affect the agricultural sector through losses in livestock productivity, mortality and international trade restrictions (Tambi et al., 2006; Jores et al., 2013). The members of this cluster include: *M. mycoides* subsp. *mycoides* (*Mmm*) the causative agent of contagious bovine pleuropneumonia (CBPP), *M. capricolum* subsp. *capripneumoniae (Mccp)* the agent of contagious caprine pleuropneumonia (CCPP), *M. capricolum* subsp. *capricolum (Mcc), M. leachii*, and *M. mycoides* subsp. c*apri* (*Mmc*). CBPP and CCPP are major livestock diseases, also called transboundary animal diseases (TADs) which negatively impact the agricultural sector specifically in developing countries through livestock product trade, food-supply reduction and decrease productivity (FAO, 2016). Furthermore, these diseases remain a threat to disease-free countries (Tambi et al., 2006); therefore, better control measures are needed (Jores et al., 2013).

The *Mycoplasma* are resistant to many antimicrobials as they lack a cell wall. The current antimicrobials widely used to treat infections with *Mycoplasma* are limited to tetracyclines, macrolide-lincosamide-streptogramin-ketolide antibiotic group and fluoroquinolones (Arjoon et al., 2012; Kama-Kama et al., 2016). Therefore, there is need to develop new antimicrobials from plant origin because of their availability and affordability and because they might provide new pathways for interfering with mycoplasma survival.

*Solanum aculeastrum* belongs to the genus of *Solanum* in the family of *Solanaceae*. It is used as a medicinal plant in many African countries (Beaman-Mbaya and Muhammed, 1976; Odhiambo et al., 2011). In Kenya, *S. aculeastrum* is used traditionally to treat sexually transmitted diseases by the Luo and Kuria communities (Wanyonyi et al., 2002; Owuor et al., 2012). *Piliostigma thonningii*, on the other hand, belongs to the genus *Piliostigma* in the family of *Fabaceae*. Its roots and twigs are used as remedy for dysentery, fever, respiratory ailments and snakebites, leaves are edible, and chewed by Maasai to relieve thirst (Jimoh and Oladiji, 2005; Bako et al., 2005; Kama-Kama et al., 2016).

Following our latest work on the preliminary screening of 152 extracts from 20 selected medicinal plants from the Kenyan flora (Kama-Kama et al., 2016), the current work focuses on investigating the phytochemistry and the antimycoplasmal activities of compounds from the berries of *S. aculeastrum* and the stem bark of *P. thonningii*. The crude extracts from these two plants showed interesting activities against selected *Mycoplasma mycoides* strains in previous studies, hence the motivation to carry out this research (Kama-Kama et al., 2016).

Growth of *Mycoplasma* has been determined by color changing units (CCU), measuring a decrease in pH after sufficient growth by a change in color (red to yellow) of the pH indicator (Stemke and Robertson, 1982). In this paper, we have used both CCU and a flow cytometric (FCM) method (Assunção et al., 2005, 2006) to follow growth of *Mycoplasma* and its inhibition by plant compounds.

## Experimental Section

### General Experimental

Column chromatography was carried out using Merck silica gel 40 (70–230 mesh) as the stationary phase. Analytical thin layer chromatography (TLC) was carried out using Merck pre- coated 60 F254. Compounds were visualized under UV light at 254 or 365 nm, followed by placing the plates in iodine tanks to view spots that were inactive under the two UV wavelengths used. 1D and 2D Nuclear Magnetic Resonance (NMR) spectra were recorded in deuterated chloroform (CDCl_3_) on a 400 MHz Bruker AVANCE NMR instrument at room temperature. Chemical shifts were expressed in ppm and referenced against the solvent resonances at 7.26 and 77.23 ppm for ^1^H and ^13^C NMR, respectively. Electron ionization mass spectroscopy (EIMS) spectra were recorded on 70 eV, SSQ 710 MAT mass spectrometer.

### Plant Material

The plant materials (about 1kg of *S. aculeastrum* berries and stem bark of *P*. *thonningii*, each) were collected from Migori and Kisumu counties (Kenya) in June and July 2014, respectively. The plant materials were immediately transported in open polythene bags from the collection site to the Department of Chemistry, University of Nairobi. The identification of these plants was done by Mr. Patrick Chyalo Mutiso, a plant taxonomist at the University of Nairobi herbarium, School of Biological Sciences (SBS), College of Biological and Physical Sciences (CBPS) where voucher specimens (*Fmk2012/14* and *Fmk2012/9*) were deposited.

The berries of *S. aculeastrum* and the stem bark of *P. thonningii* were dried at room temperature under the shade away from direct sunlight for 3 weeks. The plant materials were then ground into fine powder using a MM 20 grinder (Wiley laboratory mill, standard model No. 2, Arthur H. Thomas Company).

### Extraction with 50% MeOH in CH_2_Cl_2_ and Pure MeOH

Powdered ground berries of *S. aculeastrum* and stem bark of *P. thonningii* (1 Kg, each) were extracted by cold percolation using a mixture of 50% of MeOH in CH_2_Cl_2_ and the resultant extracts filtered under pressure. The solvent was removed *in vacuo* using a rotary evaporator resulting in 94 and 150 g of the crude extracts from *S*. *aculeastrum* and *P*. *thonningii* respectively. The insoluble residues from *S*. *aculeastrum* (see the earlier extractions) was soaked into MeOH and left overnight. The extracts obtained were filtered and concentrated as earlier described yielding 20.6 g of MeOH extract.

### Isolation of the Compounds from the Berries of *S. aculeastrum* and the Stem Bark of *P. thonningii*

About 50 g of crude 50% MeOH–CH_2_Cl_2_ extracts from the berries of *S. aculeastrum* was adsorbed onto 50 g of silica gel, left to dry and loaded on a column packed as follows: 300 g of silica gel was packed into a column using 30% of dichloromethane (CH_2_Cl_2_) in *n*-hexane (*n*-C_6_H_14_) and left standing overnight. Elution was done with *n*-C_6_H_14_ containing an increasing percentage of CH_2_Cl_2_. One hundred and sixty fractions (500 ml each) were collected and combined on the basis of the similarities of their TLC profiles. This resulted in a total of 11 fractions that were labeled as *F*_A_ to *F*_K_. Fraction D from the main column yielded 48 mg of the first compound, (17-(5-ethyl-3-hydroxy-6-methylheptan-2-yl)-2,3,6,7,8,9,10,11,12,13,14,15,16,17-tetradecahydro-10,13,14-tri- methyl-3-oxo-1H-cyclopenta[a]phenanthren-6-yl benzoate) (**1**), while fraction B yielded 8.7 mg of triterpene lupeol (**2)** and 12 mg of compound **3**. Fraction C yielded 32.5 mg of compound **4** while compound **5** and **6** were isolated from fractions *D* and *F* to yielded 7 mg and 11 mg respectively. The methanol extract (20 g) from the berries of this plant was chromatographed on a silica gel column (200 g) starting with 100% EtOAc and increasing the polarity with MeOH to yield 45 mg of the last compound (**7**), (25R)-3β-*O*-α-L-rhamnopyranosyl-(1→2)-*O*-[α-L-rhamnopyranosyl-(1→4)]-β-D-glucopyranosyloxy-22α-*N*-spirosol-5-ene (**7**).

As for the isolation of compounds from the CH_2_Cl_2_/MeOH extract from the stem bark of *P. thonningii*, 100 g of crude extracts from this plant was adsorbed onto 100 g of silica gel, left to dry and loaded on a column packed as follows: One kilogram of silica gel was used to pack a column under 80% CH_2_Cl_2_ in *n*-C_6_H_14_ and left standing overnight. The extract was loaded onto the pre-packed column and eluted initially with 2000 ml of 80% CH_2_Cl_2_ in *n*-C_6_H_14_ increasing the polarity with CH_2_Cl_2_ up to 100% CH_2_Cl_2_, followed by 1% MeOH in CH_2_Cl_2_ with increased polarity up to 20% MeOH in CH_2_Cl_2_. This column yielded 350 mg of compound **8.**

### Preparation of Compounds for Antimycoplasmal Activity Tests

The pure compounds (5 mg) were reconstituted into 1 ml of dimethyl sulfoxide (DMSO) to make a stock solution of 5 mg/ml. The mixture was prepared by vortexing to ensure homogenization of the solution and 20 μl from the stock solution (containing 100 μg of the compound) were used as a starting concentration for the antimycoplasmal activity tests.

### Bacterial Strains and Culture Conditions

All laboratory manipulations with *Mycoplasma* were carried out under BSL2 conditions (Mwirigi et al., 2016). Two *Mycoplasma mycoides* subsp. *capri* (211/94 and 95010), two *Mycoplasma mycoides* subsp. *Mycoides* (Afadé and Gladysdale), and one *Mycoplasma* capricolum subsp *capricolum* (6443-90) (**Tables 2, 3**) were cultured in Pleuropneumonia Like-Organism (PPLO) broth (Difco^TM^ PPLO Broth) media prepared as follows: 21 g of PPLO was dissolved in 700 ml of distilled water and autoclaved for 15 min at 121°C. The mixture was cooled in a water bath to 55°C and supplemented with phenol red (Carl Roth GmbH) to a final concentration of 3%, 200 ml horse serum (Sigma), 0.25% of glucose (Carl Roth GmbH), 0.15% of penicillin G (Carl Roth GmbH) and 0.25% of thallium acetate (Carl Roth GmbH). A 48 well-plate was used to grow *Mycoplasma* strains where they were incubated at 37°C for a minimum period of seven days. Growth of *Mycoplasma* cells was determined by color change from red to yellow (Stemke and Robertson, 1982), as a result of pH change due to the growth. Stock cultures of *Mmc, Mmm*, and *Mcc* were grown to a density of approximately 1–3 10^6^ cells per ml as measured by both the colony forming units (CFU) method (Stemke and Robertson, 1982) and the use of FCM (Assunção et al., 2006) after cryopreservation in a freezer at -80°C for further antimycoplasmal activity tests.

### Analysis of Mycoplasma Using Flow Cytometer

**FIGURE 1:**
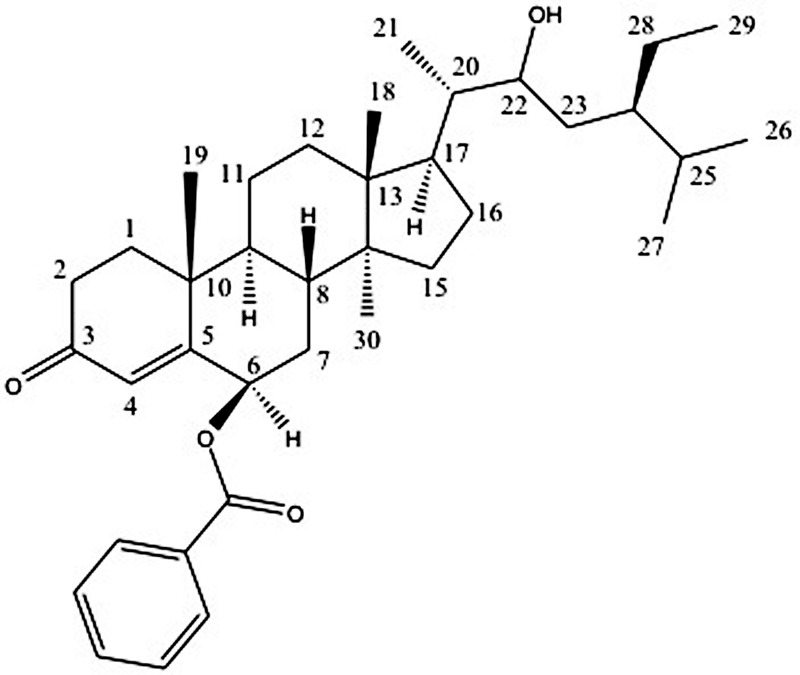
Structure of compound 1.

Growth of *Mycoplasma* (*Mmm, Mmc*, and *Mcc*) cells in culture were followed using FCM (Assunção et al., 2006). The modern FACs Canto II (BD Biosciences) with a 488 nm solid-state laser was used. To minimize the extent of contamination with small particles that could show up as *Mycoplasma* on the flow cytometer, a solution of 0.9% saline was filtered and used as sheath fluid and for sample dilution. Only forward scatter (FSc) and side scatter (SSc) were recorded and the number of events was recorded after every 30 seconds when its rate was constant. The concentration of *Mycoplasma* (cells per microliter) was calculated as the number of events acquired within the FSc/SSc window correlating with *Mycoplasma* parameters (see **Figure 1**) in one minute, and divided by the flow rate (12 μl/min). BD Diva software was used to analyze the data.

### Antimicrobial Susceptibility Testing

The broth microdilution method as described by Arjoon et al. (2012) was used to characterize the *in vitro* antimycoplasmal activity of compounds **1, 3, 4, 7**, and **8** against the growth of the *Mycoplasma* strains used in this study. Briefly, a stock solution of 5 mg was reconstituted into 1 ml of DMSO (5 mg/ml). From the stock solution, 20 μl was used and made up to 1 ml with cell culture (giving a concentration of 100 μg/ml) for the antimycoplasmal test. Two control samples were tested in parallel, consisting of the following: media plus *Mycoplasma* (negative control) and media plus *Mycoplasma* plus tetracycline (positive control). The kinetics of the bioassays were determined by monitoring the concentrations of the *Mycoplasma* cells used for the inhibition assays from the first day – to the seventh day (day 0– day 6). The cultures were also monitored for color change (from red–yellow) due to the change of pH, an indication of the growth of *Mycoplasma* cells. No color change meant that the compounds in the well prevented growth of *Mycoplasma*. Data were analyzed using Graphprism software version 6.0.

### Determination of the Minimum Inhibitory Concentration (MIC)

Active compounds were serially diluted in order to determine the minimum inhibitory concentration (MIC), which was defined as the lowest concentration that inhibited the growth of different *Mycoplasma* strains for an incubation time of seven days. The MICs of all the active compounds were determined by broth microdilution as previously described by Arjoon et al. (2012) starting from 100 to 5 μg/ml. The experiment was repeated thrice for each assay and mean was recorded. The standard error of mean was obtained at *P* < 0.05.

## Results

Chromatographic purification of compounds from the 50% MeOH in CH_2_Cl_2_ extract of the berries of *S. aculeastrum* led to the isolation of the following compounds; a β-sitosterol derivative;(17-(5-ethyl-3-hydroxy-6-methylheptan-2-yl)-2,3,6,7,8,9,10,11,12,13,14,15,16,17-tetradecahydro-10,13,14-trimethyl-3-oxo-1H-cyclopenta[a]phenanthren-6-yl benzoate) (**1**) and the commonly known triterpene, lupeol (**2**), two long-chain fatty alcohols namely lauryl alcohol (**3**) and undecyl alcohol (**4**); two long-chain fatty acids namely myristic acid (**5**) and nervonic acid (**6**) while the MeOH extract of the same plant material led to the isolation of a known triglycosidic steroidal alkaloid; (25R)-3β-*O*-α-L-rhamnopyranosyl-(1→2)-*O*-[α-L-rhamnopyranosyl-(1→4)]-β-D-glucopyranosyloxy-22α-*N*-spirosol-5-ene (**7**). Using the same method, the CH_2_Cl_2_/MeOH extracts from the stem bark of *Piliostigma thonningii* led to the isolation of a new diglycoside furan (**8**). The structures of these compounds (**1,3**–**8**) were determined on the basis of spectroscopic techniques while the triterpene, lupeol (**2**) was identified by comparison with an authentic sample. Both NMR and MS data of compounds 1 and 8 are presented in the Supplementary Material. The antimycoplasmal activity test of the isolated compounds showed that compounds **4** and **7** exhibited moderate activities as compared to the control (tetracycline). The lowest MIC value was 50 μg/ml for compound **7** against *Mmm*.

### Compounds from the Berries of *S. aculestrum*

#### Compound 1

Compound **1** was isolated as white crystals, with melting point (mp) above 210°C. The HRMS (Supplementary 1) of this compound showed an ion peak at 563.4011 (calculated mass was of 562.40) corresponding to a molecular formula of C_37_H_54_O_4_. Most of the peaks in the ^1^H and ^13^C NMR spectra of this compound match those of β-sitosterol (Quillez et al., 2003) with an additional benzoate substituent at position C-6, as shown in **Figure 1**.

The ^1^H NMR (Supplementary 1) indicated seven characteristic methyls signals at δ_H_ 0.62 (3H, *br, s*), δ_H_ 0.83 (3H, *m*), δ_H_ 0.91 (3H, *m*), δ_H_ 0.93 (3H, *m*), δ_H_ 0.96 (3H, *d, J* = 6.72), δ_H_1.10 (3H, *d, J* = 5.91 Hz), and δ_H_1.31 (3H, *m*). Six doublets were observed at δ_H_ 0.96 (3H, *d, J* = 6.72 Hz), δ_H_ 1.10 (3H, *d, J* = 5.91 Hz), δ_H_ 1.20 (1H, *d, J* = 4.85 Hz), δ_H_ 2.27 (1H, *d, J* = 10.89 Hz), δ_H_ 3.73(1H, *d, J* = 0.69 Hz), and δ_H_ 5.70 (1H, *d, J* = 2.34 Hz). The doublet at δ_H_ 5.70 (1H, *d, J* = 2.34 Hz) at carbon C-4 is a characteristic of olefinic protons. The Characteristic aromatic protons were observed at δ_H_ 8.06 (2H, *dd, J*_1_ = 8.10 Hz, and *J_2_* = 1.37 Hz), δ_H_ 7.56 (1H, *m*), and δ_H_ 7.45 (2H, *t, J_1_* = *J_2_* = 7.73 Hz). The protons H-6 and H-22 were downfield resonating at δ_H_ 4.69 and at δ_H_ 3.75 because of the oxygenation.

The ^13^C NMR (Supplementary 1) spectrum of the compound **1** showed 37 carbon signals, 30 of which are from the sitosterol base skeleton, and 7 from the benzoate group. The carbon C-3 at δ_C_ 200.1 is characteristic of a keto carbon while carbon C-1′ at δ_C_ 166.4 corresponds to an ester carbonyl carbon. The HMBC (Supplementary 1) experiment showed cross peaks between H-6 resonating at δ_H_ 4.69 and C-5 (δ_C_ 161.0) and C-1′ (δ_C_ 166.4) confirming the placement of the benzoate moiety at C-6. Furthermore, the correlation observed between H-22 at δ_H_ 3.75 with C-21 resonating at d_C_ 17.6 also confirms the placement of the hydroxyl group at C-22. The stereochemistry of the base skeleton, the β-sitosterol is biogenetically known where the methyls CH_3_-19 and CH_3_-18 are on the same side while H-9 is on the opposite side. The ^1^H NMR data showed that H-6 was coupling with H-8 (each 1H, *J* = 3.41 Hz) suggesting that they are on the opposite direction of the β-sitosterol skeleton. Similarly, H-8 was also coupling with H-9 (each 1H, *J* = 4.71 Hz) suggesting that H-8 and H-9 are on the opposite side (Garbisch and Griffith, 1968). This was confirmed by the NOESY (Supplementary 1) experiment, which showed a correlation between H-9 and H-6 suggesting they are on the same side. The NOESY 1) experiment further showed that there was a correlation between H-21 and H-22 (1H each, *J* = 10.29 Hz) suggesting they are on the same side. However, the NOESY experiment did not show any correlation between the methyls CH_3_-18 and CH_3_-30 suggesting that they are in *trans* configuration.

Based on these data coupled with comparison of these spectra with those in the literature, compound 1 was identified as (17-(5-ethyl-3-hydroxy-6-methylheptan-2-yl)-2,3,6,7,8,9,10,11,12,13,14,15,16,17-tetradecahydro-10,13,14-trimethyl-3-oxo-1H-cyclopenta[a]phenanthren-6β-yl benzoate). This is the first report of a derivative of β-sitosterol with a benzoate substituent at C-6 position. Therefore, compound **1** was identified as a novel β-sitosterol derivative namely β-sitosterol-6β-benzoate. The 1H (400 MHz) and 13C NMR data for compound **1** are presented in **Table 1** while its structure is shown in **Figure 1**.

**Table 1:** ^1^H (400MHz) and ^13^C NMR data for compound **1** (CDCl_3_).

Carbon	σC	HMQC (σH)	DEPT	HMBC
1	38.8	2.14; 1.47 (2H, *m*)	CH_2_	C (δ_C_ 200.1; 36.2)
2	36.2	1.87; 1.62 (2H, *m*)	CH_2_	C (δ_C_ 200.1; 123.6)
3	200.1	–	C	C (δ_C_ 53.1; 39.3)
4	123.6	5.71 (1H, *d, J* = 2.34 Hz)	CH	
5	161.0	–	C	
6	78.9	4.69 (1H, *ddd, J_1_* = 10.81 Hz; *J_2_*= 10.70 Hz; *J_3_*= 4.71 Hz)	CH	C (δ_C_ 161.0; 166.4)
7	41.4	1.28 (1H, *m*)	CH	
8	42.6	1.73 (1H, *m)*	CH	
9	54.9	2.06 (H, *m*)	CH	
10	39.3	–	C	
11	21.7	1.80; 1.63 (2H, *m*)	CH_2_	
12	42.8	1.73 (1H, *m*)	CH	
13	45.1	–	C	
14	53.1	–	C	
15	23.6	1.47 (1H, *m*); 1.28 (1H, *t, J_1_*= *J_2_*= 3.82 Hz)	CH_2_	
16	30.0	1.25; 1.04 (2H, *m*)	CH_2_	
17	60.0	2.27 (1H, *d, J* = 10.89 Hz)	CH	C (δ_C_ 31.9; 12.3; 51.0)
18	11.8	0.91 (3H, *m*)	CH_3_	
19	20.5	0.93 (3H, *m*)	CH_3_	
20	31.8	1.73 (1H, *brs*) (*d, J* = 10.30 Hz)	CH	
21	17.6	0.83(3H, *s*),	CH_3_	
22	71.0	3.75 (1H, *d, J* = 10.29 Hz)	CH	C (δ_C_ 17.6; 31.8)
23	26.1	2.01; 1.23 (2H, *m*)	CH_2_	
24	51.0	2.26 (1H, *m*)	CH	
25	28.7	1.70 (1H, *m*)	CH	
26	17.4	0.85 (3H, *m*)	CH_3_	
27	14.7	0.96 (3H, *d, J* = 6.72 Hz)	CH_3_	
28	22.5	1.62; 1.54 (2H, *m*)	CH_2_	
29	12.5	0.97 (3H, *m)*	CH_3_	
30	12.3	0.62(3H, *br, s*)	CH_3_	
1″	166.4	–	C	–
2″	130	–	C	–
3″	129.6	8.06 (2H, *dd, J*_1_= 8.10 Hz; *J*_2_= 1.37 Hz)^∗^	CH	C (δ_C_ 166.5; 132.9; 129.6)
4″	128.4	7.45 (2H, *t, J_1_*= *J_2_*= 7.73 Hz)^∗∗^	CH	C (δ_C_ 130.6; 128.4)
5″	132.8	7.56 (1H, *m)*	CH	C (δ_C_ 129.6)
6″	128.4	^∗∗^	CH	C (δ_C_ 130.6; 128.4)
7″	129.6	^∗^	CH	C (δ_C_166.5; 132.9; 129.6)

#### Compound 7

Compound **7** was isolated as light brown crystals, with a mp of 200–205°C. The ^1^H-NMR and ^13^C-NMR spectra of this compound was comparable with that of a steroidal alkaloid glycoside, solasodine triglycoside (Radeglia et al., 1977; Ripperger and Schreiber, 1981; Murakami et al., 1985; Ripperger and Himmelreich, 1994; Ripperger, 1995; Ripperger and Porzel, 1997; Vázquez et al., 1997). The HRMS showed an intense ion peak at *m*/*z* 870 (calculated 869.07) corresponding to a molecular formula C_45_H_74_0_15_N.

Additional peaks were evident at *m*/*z* 724 and 578 attributed to the loss the first and the second molecules of deoxyhexose units respectively. This fragmentation pattern was characteristic of a triglycoside steroidal alkaloids previously characterized from a number of species belonging to the genus *Solanum* including, the seeds of *S. robustum* (Ripperger and Schreiber, 1981) and the berries of *S. aculeastrum* (Wanyonyi et al., 2003).

Besides these two compounds, two long-chain fatty alcohols namely; lauryl alcohol (**3**) and undecyl alcohol (**4**); two long-chain fatty acids namely; myristic acid (**5**) and nervonic acid (**6**) were also isolated. This is the first report of these compounds from *S. aculeastrum*. The identification of these four compounds was done by comparing the generated data (^1^H NMR and ^13^C NMR) with those in the literature (Davidson and Contrill, 1985; Boven et al., 1997; Bond, 2003).

### Compounds from the Stem Bark of *P. thonningii*

#### Compound 8

**FIGURE 2:**
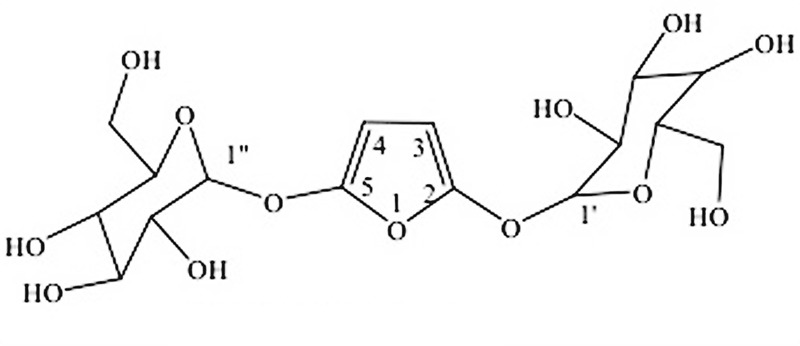
Structure of compound 8.

Compound **8** was isolated as amorphous crystals. HRMS (Supplementary 2) showed an ion peak at *m/z* 423 (calculated mass was 424) corresponding to a molecular formula C_16_H_24_O_13_. The compound was identified as a furan diglycoside. The ^1^H NMR (Supplementary 2) spectra showed four doublets, two of which were attributed to the olefinic protons, and two to anomeric protons. The two doublets resulting from the olefinic protons appeared at δ 6.02 (1H, *dd, J*_1_ = 9.58 Hz, *J*_2_ = 3.25 Hz) and δ 6.22 (1H, *dd, J*_1_ = 10.11 Hz, *J*_2_ = 1.71 Hz) assigned to C-3 and C-4, of the furan ring, respectively. However, the doublets resulting from the two-downfield shifted anomeric protons appeared at δ 5.17 (1H, *dd, J*_1_ = 7.21 Hz, *J*_2_ = 3.16 Hz) and at δ 5.68 (1H, *br, s*) and were placed at C_1_′ and C_1_″, respectively. The ^13^C NMR (Supplementary 2) showed 16 carbons assigned to a substituted furan ring, with two glucose molecules at C-2 and C-5. The HMBC (Supplementary 2) experiments showed a correlation between H-3 and C-2; and between H-1′ and C-3. Similarly, the HMBC showed a correlation between H-1″ with C-5 confirming the placement of the two glycosides on C-2 and C-5 positions of the furan ring. Based on the above spectroscopic data and comparison with literature values, it was evident that this is the first time this compound has been characterized from nature. The ^1^H (400 MHz) and ^13^C NMR data for compound **8** are presented in **Table 2** while its structure is shown in **Figure 2**.

**Table 2:** ^1^H (400MHz) and ^13^C NMR data for compound **8** (MeOD).

Carbon	σC	HMQC (σH)	DEPT	HMBC
1	-	–	–	–
2	155.8	–	C	
3	138.7	6.02 (1H, *dd, J*_1_= 9.58 Hz, *J*_2_= 3.25 Hz)	CH	C (155.9; 73.5)
4	126.4	6.22 (1H, *dd, J_1_*= 10.11 Hz; *J_2_*= 1.71 Hz)	CH	C (97.1; 75.7; 70.2)
5	154.8	–	C	
1′	102	5.17 (1H, *dd, J*_1_= 7.21 Hz, *J*_2_= 3.16 Hz)	CH	C (138.7; 70.2)
2′	77.4	3.16 (1H, *m*)	CH	
3′	75.7	4.55 (1H, *dd, J*_1_= 7.99 Hz, *J*_2_= 1.67 Hz)	CH	
4′	73.4	3.16 (1H, *m*)	CH	
5′	70.2	3.03 (1H, *m*)	CH	
6′	62	3.67; 3.45 (2H, *m*)	CH_2_	
1″	97.1	5.68 (1H, *br, s*)	CH	C (77.1; 73.5)
2″	76.9	4.61 (1H, *d, J* = 7.47 Hz)	CH	
3″	73.5	3.14 (1H, *m*)	CH	
4″	74.3	3.69 (1H, *m*)	CH	
5″	70.6	3.98 (1H, *m*)	CH	
6″	61.9	3.01(1H, *m*)	CH	

### Determination of the Antimycoplasmal Activity Tests of Pure Compounds Using Flow Cytometer

**FIGURE 3:**
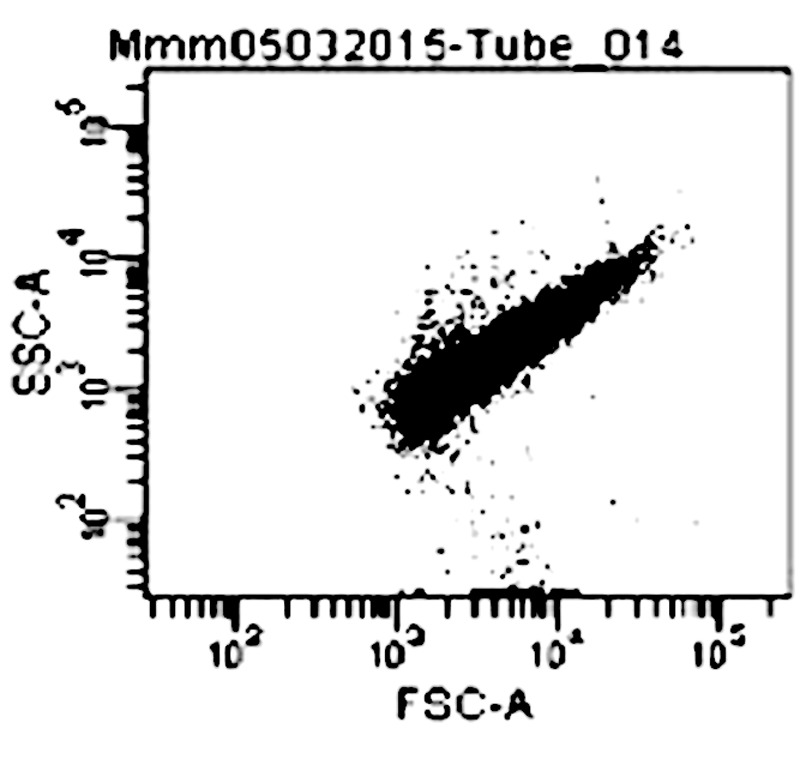
Dot plot (forward versus 90° scatter signal) of *M. mycoides mycoides* on Flow cytometer Keys: FSc (Forward scatter); SSc (Side scatter); Mmm (Mycoplasma mycoides subsp. mycoides).

Compounds **1, 3, 4, 7**, and **8** were tested against the growth of five strains of *Mycoplasma* (two stains of *Mmm*, two strains of *Mmc* and one strain of *Mcc*) together with two controls which included: media plus *Mycoplasma* (negative control), and media plus *Mycoplasma* plus tetracycline (positive control). The FCM profile analysis of *Mycoplasma* is shown in **Figure 3**.

The results of the antimycoplasmal activity tests of the pure compounds showed that only compounds **4** and **7** inhibited the growth of *Mmm* (Afadé and Gladysdale) and *Mmc* (211/94 and 95010) for 3 days only. The inhibition was observed as a delay of the growth and a partial reduction of maximum concentration. The growth of the selected *Mycoplasma* strains was clearly observed when the color of the media (PPLO) changed from red to yellow due to the change of pH as well as the increase in the concentration of the *Mycoplasma* strains used. However, it is important to note that even in the case of the inhibition control with tetracycline, where no color change was observed, the concentration of the *Mycoplasma* as measured by flow cytometry did also increase from day 3, although the concentration remained lower as compared to that of the *Mycoplasma* treated with the plant compounds.

The results of the antimycoplasmal activity using CCU only are presented in **Table 3**. We have in this case presented only two strains (one *Mmm* and one *Mmc*)

**Table 3:** Antimycoplasmal activities using color change units (CCU) method.

	*Mmm* (Afadé)				*Mmc* (211/94)			
	Controls		Compounds		Controls		Compounds	
Days	*Mmm* only	*Mmm*+ Tetracyline	4	7	*Mmc* only	*Mmc* + Tetracylin	4	7
0	–	–	–	–	–	–	–	–
1	–	–	–	–	+	–	–	–
2	+	–	–	–	+	–	–	-
3	+	–	–	–	+	–	–	+
4	+	–	–	+	+	–	+	+
5	+	–	+	+	+	–	+	+
6	+	–	+	+	+	–	+	+

*Keys: *Mmm* (*Mycoplasma mycoides* subsp.*mycoides*); *Mmc* (*Mycoplasma mycoides* subsp.*capri*); Med (Media only); Tetra (Tetracycline); **4** (Compound **4**); **7** (Compound **7**); – (Red); +(Yellow).*

**FIGURE 4:**
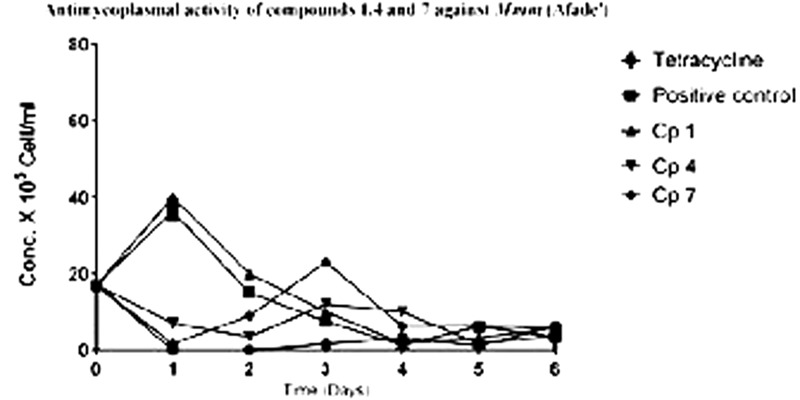
Antimycoplasmal activity tests of compounds **1, 4**, and **7** against the growth of Mmm (Afade) Keys; Cp 1 (compound l); Cp4 (compound **4**); Cp 7 (compound **7**).

**FIGURE 5:**
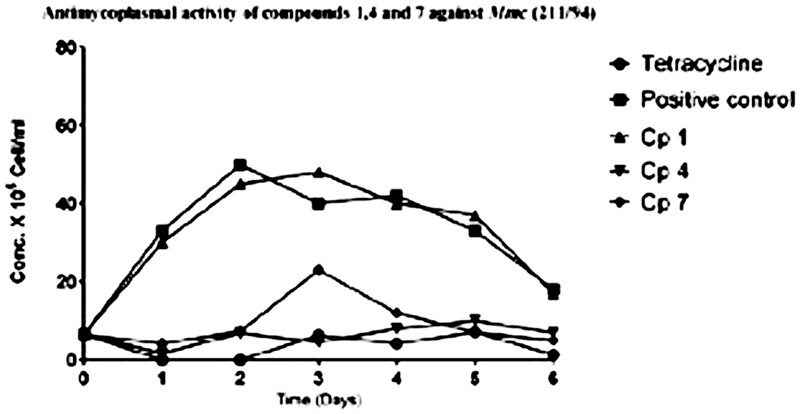
Anlimycoptasmal activity tests of compounds **1, 4**, and **7** against the growth of Mmc (211/94) Keys: Cp I (compound **1**); Cp 4 (compound **4**); Cp 7 (compound **7**).

The ability of the pure compounds to inhibit the growth of *Mycoplasma* was monitored for 6 days and the results of the kinetics of antimycoplasmal activity tests determined by the use of the FCM are presented below in **Figures 4, 5**. The summary of the antimycoplasmal activity tests of the isolated compounds is presented in **Table 4**.

**Table 4:** The antimycoplasmal potencies of isolated compounds.

Compounds	*Mmm*		*Mmc*		*Mcc*
	Afadé	Gladysdale	211/94	95010	6443-90
1	–	–	–	–	–
2	NT	NT	NT	NT	NT
3	–	NT	–	NT	NT
4	+	+	+	+	–
5	NT	NT	NT	NT	NT
6	NT	NT	NT	NT	NT
7	+	+	+	+	–
8	–	–	–	–	–
Controls
Media+*Mycoplasma* (positive control)	–	–	–	–	–
Media+*Mycoplasma*+Tetracycline (negative control)	+	+	+	+	+

*Keys: NT (not tested); + (active); – (not active); *Mmm* (*Mycoplasma mycoides* subsp. *mycoides*); *Mmc* (*Mycoplasma mycoides* subsp. *capri*); *Mcc* (*Mycoplasma capricolum* subsp. *capricolum*).*

### Determination of the MICs of the Pure Compounds

Minimum inhibitory concentrations were regarded as the lowest concentration of the pure compound able to inhibit the growth of *Mycoplasma* for a period of 7 days. However, in this case, there was growth on the third day meaning that there was only a delay in growth of *Mycoplasma* but not complete inhibition of the *Mycoplasma*. The results of the MICs are presented in **Table 5**.

**Table 5:** Mean values of minimum inhibitory concentration (MICs) of the pure compounds.

Compounds	Mean of MICs (±SEM) against *Mmm* in μg/ml	Mean of MICs (±SEM) against *Mmc* in μg/ml
**4**	100	100
**7**	50	100
Control
Tetracycline	5	5

## Discussion

The results from this study showed that compounds **4** and **7** exhibited moderate antimycoplasmal activities as compared to the control, tetracycline. The lowest minimum inhibitory concentration of active compound, **7**, was 50 μg/ml against *Mmm*.

Although the crude extracts from these two plants showed very good activities against the growth of *Mycoplasma* strains (Kama-Kama et al., 2016), these activities could not be observed with pure compounds. This could be most probably attributed to synergistic effects of all or some of the compounds occurring in these plants. The activities observed in the crude extracts and not in the isolated compounds could also be due to minor compounds which were not isolated in the current study.

The antimycoplasmal activity of compound **4** observed in this study could be attributed to the fact that it is a long chain fatty alcohol which in previous studies is known to have antibacterial activities (Kubo et al., 1995; Mukherjee et al., 2013). According to Kubo et al. (1995) the activity of long chain fatty alcohols against selected Gram-positive bacteria suddenly dropped off from C_13_ but the MICs obtained from their investigation showed that long fatty alcohol with a C_12_ exhibited a better MIC value than C_11_. However, the results from our study showed that lauryl alcohol, a C_12_ long chain fatty alcohol exhibited moderate activity while undecyl, a C_11_ long fatty alcohol was inactive against *Mycoplasma mycoides* strains tested. The MIC value of compound **4** was also higher (100 μg/ml) as compared to the control, tetracycline (5 μg/ml). This was also observed in previous studies on other plant compounds (Kubo et al., 1995) where the MIC value ranged from 12.5 to 25 μg /ml. This could be because *Mycoplasmas* are different from other bacteria strains as they have a much smaller genome (Westberg et al., 2004).

Compound **7** is a steroidal alkaloid glycoside and previous reports on its biological activity showed that this compound exhibited antimicrobial as well as anticancer activity (Wanyonyi et al., 2002; Shabana et al., 2013). The minimal activity exhibited by **7** could be explained by the fact that it is a steroidal alkaloid glycoside and contains in its structure so many OH groups that could have been hydrolyzed before reaching the target and consequently losing its activity. Nevertheless, it is clear that the pure compounds exhibited weak activity as compared to the crude extracts (Kama-Kama et al., 2016).

The use of both FCM and CCU methods for the antimycoplasmal activity tests has proven to be more efficient that than the CCU alone. The color change method only tells us when the mycoplasma have grown enough to make the medium acidic, while the FCM method allows us to see growth before that time or to see weak growth that is not able to change the pH. Using FCM, the antimycoplasmal activity test of compounds **4** and **7** showed that the two compounds delayed the growth of the *Mycoplasma* and to lower maxima than the control samples without compound or samples with compounds **1, 3**, or **8** that showed no activity. While tetracycline was a much better inhibitor than the plant compounds, we did find using FCM that there still was weak growth with the antibiotic after a number of days. But this could not be observed with the CCU method. The latter method did confirm that the two compounds, **4** and **7**, had activity against *Mmm* and *Mmc* as they delayed the change in pH-induced color change.

The weak increase in *Mycoplasma* concentration 3 days after tetracycline treatment could have several causes; one is that tetracycline at this concentration may not kill all *Mycoplasma*. *In vivo* experiments have shown that the antibiotic does reduce mycoplasmemia and fever in *Mmm*-infected cattle, but a resurgence of fever occurred after several days (Schieck et al., 2014). Alternatively, these *Mycoplasmas* might have developed resistance to tetracycline. Previous works also confirmed that *Mycoplasma* have developed resistance to tetracycline (Dégrange et al., 2008).

Combining FCM and CCU methods in the antimycoplasmal activity test gave more detailed information and could be used for slow growing bacteria such as *Mycoplasma* in order to quickly establish the resistance of these organisms to the antimicrobial agents.

## Conclusion and Recommendation

From the berries of *S. aculeastrum*, a new β-sitosterol benzoate namely; (17-(5-ethyl-3-hydroxy-6-methylheptan-2-yl)-2,3,6,7,8,9,10,11,12,13,14,15,16,17-tetradecahydro-10,13,14-trimethyl-3-oxo-1H-cyclopenta[a]phenanthren-6β-yl benzoate) (**1**) and six known compounds; lupeol (**2**) two long-chain fatty alcohols namely; lauryl alcohol (**3**) and undecyl alcohol (**4**); two long-chain fatty acids namely; myristic acid (**5**) and nervonic acid (**6**) and a steroidal alkaloid triglycoside; (25*R*)-3β-*O*-α-L-rhamnopyranosyl-(1→2)-O-[α-L-rhamnopyranosyl-(1→4)]-β-D-glucopyranosyloxy-22αN-spirosol-5-ene ion (**7**), were isolated. Furthermore, the stem bark of *Piliostigma thonnignii* yielded a new diglycoside furan (**8**). The antimycoplasmal activity tests of these compounds against the growth of selected *Mycoplasma mycoides* strains showed that compounds **4** and **7** had moderate activities with the lowest mean MIC value of 50 μg/ml for **7** against *Mmm*. The activities of these compounds were lower as compared to those of the crude extracts. Structural modification of these compounds could increase their antimycoplasmal activities. Alternatively, isolation and purification of compounds from these plants could yield additional compounds with interesting activity. These compounds should also be tested against other strains of bacteria, as well as their safety profiles ascertained.

Flow cytometry measurement of *Mycoplasma* concentrations allows one to obtain a more precise growth kinetics and measure growth before a color change occurs. However, the technique needs the right equipment and is more labor intensive.

## Author Contributions

FK-K, JM, LO, and JN conceived and designed the experiments. FK-K performed the experiments. FK-K, LO, MI, and SY analyzed the data. FK-K, LO, SY, JNG, NM, JM, GO, and JN wrote the paper.

## Conflict of Interest Statement

The authors declare that the research was conducted in the absence of any commercial or financial relationships that could be construed as a potential conflict of interest.

## References

[B1] Arjoon A. V. Saylor C. V. May M. (2012). *In vitro* efficacy of antimicrobial extracts against the atypical ruminant pathogen *Mycoplasma mycoides* subsp *capri*. *BMC Complement. Altern. Med.* 12:169. 10.1186/1472-6882-12-169 23031072 PMC3517410

[B2] Assunção P. Antunes N. T. Rosales R. S. Poveda C. Poveda J. B. Davey H. M. (2005). Flow cytometric determination of the effects of antibacterial agents on *Mycoplasma agalactiae, Mycoplasma putrefaciens, Mycoplasma capricolum* subsp *capricolum*, and *Mycoplasma mycoides* subsp *mycoides* large colony type. *Antimicrob. Agents Chemother.* 50 2845–2849. 10.1128/AAC.01582-05 16870783 PMC1538642

[B3] Assunção P. de la Fe C. Antunes N. T. Rosales R. S. De Galarreta C. M. R. Poveda J. B. (2006). Use of flow cytometry for enumeration of *Mycoplasma mycoides* subsp *mycoides* large-colony type in broth medium. *J. Appl. Microbiol.* 100 878–884. 10.1111/j.1365-2672.2005.02858.x 16553745

[B4] Bako S. P. Bakur M. J. John I. Bala E. I. (2005). Ethnomedicinal and phytochemical profile of some savanna species in Nigeria. *Int. J. Bot.* 1 147–150. 10.3923/ijb.2005.147.150

[B5] Beaman-Mbaya V. Muhammed S. I. (1976). Antibiotic action of *Solanum incanum* Linnaeus. *Antimicrob. Agents Chemother.* 9 920–924. 10.1128/AAC.9.6.920 945715 PMC429651

[B6] Bond A. D. (2003). On the crystal structures and melting point alternation of the n-alkyl carboxylic acids. *New J. Chem.* 28 104–114. 10.1039/B307208H

[B7] Boven M. V. Da P. Ma K. Cokela M. (1997). Content and composition of free sterols and free fatty alcohols from Jojoba oil. *J. Agric. Food Chem.* 45 1180–1184. 10.1021/jf960488g

[B8] Davidson B. C. Contrill R. (1985). Fatty acid nomenclature. *S. Afr. Med. J.* 67 633–634. 3885429 10.1002/chin.198542383

[B9] Dégrange S. Renaudin H. Charron A. Be C. (2008). Tetracycline resistance in *Ureaplasma* spp. and *Mycoplasma hominis*: prevalence in Bordeaux, France, from 1999 to 2002 and description of two *tet*(M)-positive isolates of *M. hominis* susceptible to tetracyclines. *Antimicrob. Agents Chemother.* 52 742–744. 10.1128/AAC.00960-07 18025113 PMC2224736

[B10] FAO (2016). *Economic Analysis of Animal Diseases* Vol. 18 Rome: FFAO Animal Production and Health Guidelines.

[B11] Fischer A. Shapiro B. Muriuki C. Heller M. Schneee C. Bongcam-Rudoff E. (2012). The origin of the ‘*Mycoplasma mycoides* cluster’ coincides with domestication of ruminants. *PLOS ONE* 7:e36150. 10.1371/journal.pone.0036150 22558362 PMC3338596

[B12] Garbisch E. W. Griffith M. (1968). Proton couplings in cyclohexane. *J. Am. Chem. Soc.* 480 6543–6544. 10.1021/ja01025a069

[B13] Jimoh F. O. Oladiji A. T. (2005). Preliminary studies on *Piliostigma thonningii* seeds: proximate analysis, mineral composition and phytochemical screening. *Afr. J. Biotechnol.* 4 1439–1442. 10.4314/ajb.v4i12.71459

[B14] Jores J. Mariner J. C. Naessens J. (2013). Development of an improved vaccine for contagious bovine pleuropneumonia: an African perspective on challenges and proposed actions. *Vet. Res.* 44 1–5. 10.1186/1297-9716-44-122 24359340 PMC3910389

[B15] Kama-Kama F. Midiwo J. Nganga J. Maina N. Schiek E. Kerubo L. (2016). Selected ethno-medicinal plants from Kenya with in vitro activity against major African livestock pathogens belonging to the *Mycoplasma mycoides* cluster. *J. Ethnopharmacol.* 192 524–534. 10.1016/j.jep.2016.09.034 27649681 PMC5081062

[B16] Kubo I. Muroi H. Kubo A. (1995). Structural functions of antimicrobial long-chain alcohols and phenols. *Bioorg. Med. Chem.* 3 873–880. 10.1016/0968-0896(95)00081-Q 7582963

[B17] Mukherjee K. Tribedi P. Mukhopadhyay B. Sil A. K. (2013). Antibacterial activity of long-chain fatty alcohols against mycobacteria. *FEMS Microbiol. Lett.* 338 177–183. 10.1111/1574-6968.12043 23136919

[B18] Murakami K. Ezima H. Takaishi Y. Takeda Y. Fujita T. Sato A. (1985). Studies on constituents of Solanum plants: the constituents of *S. lyratum* THUNB.II. *Chem. Pharm. Bull.* 33 67–73. 10.1248/cpb.33.67

[B19] Mwirigi M. Nkando I. Aye R. Soi R. Ochanda H. Berberov E. (2016). Veterinary immunology and immunopathology experimental evaluation of inactivated and live attenuated vaccines against *Mycoplasma mycoides* subsp. *mycoides*. *Vet. Immunol. Immunopathol.* 169 63–67. 10.1016/j.vetimm.2015.12.006 26827840

[B20] Odhiambo J. A. Lukhoba C. W. Dossaji S. F. (2011). Evaluation of herbs as potential drugs/medicines. *Afr. J. Tradit. Complement. Altern. Med.* 8(Suppl. 5) 144–151. 10.4314/ajtcam.v8i5S.20 22754068 PMC3252725

[B21] Owuor B. O. Ochanda J. O. Kokwaro J. O. Cheruiyot A. C. Yeda R. A Okudo C. A. (2012). *In vitro* antiplasmodial activity of selected Luo and Kuria medicinal plants. *J. Ethnopharmacol.* 144 779–781. 10.1016/j.jep.2012.09.045 23041700

[B22] Quillez J. Garcila-Lorda P. Salas-Salvadó J. (2003). Potential uses and benefits of phytosterols in diet: present situation and future directions. *Clin. Nutr.* 22 343–351. 10.1016/S0261-5614(03)00060-8 12880600

[B23] Radeglia R. Ripperger H. Adams G. (1977). 13C NMR spectroscopy of steroidal alkaloid. *Tetrahedron Lett.* 11 903–906. 10.1016/S0040-4039(01)92787-X

[B24] Ripperger H. (1995). Steroid alkaloid glycosides from *Solanum robustum*. *Phytochemistry* 29 1475–1477. 10.1016/0031-9422(95)00150-6 7669283

[B25] Ripperger H. Himmelreich U. (1994). Anguivine and isoanguivine steroid alkaloid glycosides from *Solanum anguivi*. *Phytochemistry* 17 1725–1727. 10.1016/S0031-9422(00)89600-4 7766005

[B26] Ripperger H. Porzel A. (1997). Steroidal alkaloid glycosides from *Solanum suaveolens*. *Phytochemistry* 46 1279–1282. 10.1016/S0031-9422(97)80027-1 9423293

[B27] Ripperger H. Schreiber K. (1981). Solanum steroid alkaloids. *Chem. Physiol.* 19 181–183.

[B28] Schieck E. Liljander A. Hamsten C. Gicheru N. Scacchia M. Sacchini F. (2014). High antibody titres against predicted *Mycoplasma* surface proteins do not prevent sequestration in infected lung tissue in the course of experimental contagious bovine pleuropneumonia. *Vet. Microbiol.* 172 285–293. 10.1016/j.vetmic.2014.04.018 24880898

[B29] Shabana M. M. Salama M. M. Ezzat S. M. Ismail L. R. (2013). In vitro and in vivo anticancer activity of the fruit peels of Solanum. *J. Carcinog. Mutagen.* 4 1–6. 10.4172/2157-2518.1000149

[B30] Stemke G. W. Robertson J. A. (1982). Comparison of two methods for enumeration of mycoplasmas. *J. Clin. Microbiol.* 16 959–961. 7153345 10.1128/jcm.16.5.959-961.1982 PMC272510

[B31] Tambi N. E. Maina W. O. Ndi C. (2006). An estimation of the economic impact of contagious bovine pleuropneumonia in Africa. *Rev. Sci. Tech.* 25 999–1011. 10.20506/rst.25.3.1710 17361766

[B32] Vázquez A. Gonzalaz G. Ferreira F. Moyna P. Kenne L. (1997). Glycoalkaloids of *Solanum commersonii* Dun. ex Poir. *Euphytica* 95 195–201. 10.1023/A:1002997616784

[B33] Wanyonyi A. W. Chhabra S. C. Mkoji G. Eilert U. Njue W. M. (2002). Bioactive steroidal alkaloid glycosides from *Solanum aculeastrum*. *Phytother. Res.* 59 79–84. 10.1016/S0031-9422(01)00424-1 11754948

[B34] Wanyonyi A. W. Tarus P. K. Chhabra S. C. (2003). A novel glycosidic steroidal alkaloid from *Solanum aculeastrum*. *Bull. Chem. Soc. Ethiop.* 17 61–66. 10.4314/bcse.v17i1.61733

[B35] Weisburg W. G. Tully J. G. Rose D. L. Petzel J. P. Oyaizu H. Yang D. (1989). A phylogenetic analysis of the mycoplasmas: basis for their classification. *J. Bactriol.* 171 6455–6467. 10.1128/jb.171.12.6455-6467.1989 PMC210534 2592342

[B36] Westberg J. Persson A. Holmberg A. Goesmann A. Lundeberg J Uhlén M. (2004). The genome sequence of *Mycoplasma mycoides* subsp. *mycoides* SC type strain PG1 T, the causative agent of contagious bovine the genome sequence of *Mycoplasma mycoides* subsp. *mycoides* SC type strain PG1 T, the causative agent of contagious bovine pleurop. *Genome Res.* 14 221–227. 10.1101/gr.1673304 14762060 PMC327097

