# Supplementary material for: Antimycoplasmal Activities of Compounds from Solanum aculeastrum and Piliostigma thonningii against Strains from the Mycoplasma mycoides Cluster
Source: Front Pharmacol. 2017 Dec 21;8:920. doi: 10.3389/fphar.2017.00920 (PMC5742823; doi:10.3389/fphar.2017.00920)
Supplement: Supplementary file 1 [file Data_Sheet_1.docx]

Supplementary information

Supplementary 1

^1^HNMR CMP 1

^13^ CNMR

COSY CMP 1

NOESY CMP 1

HSQC CMP 1

HMBC CMP 1

HRMS compound 1

Supplementary 2

NMR CMP- 8

^1^H NMR CMP-8


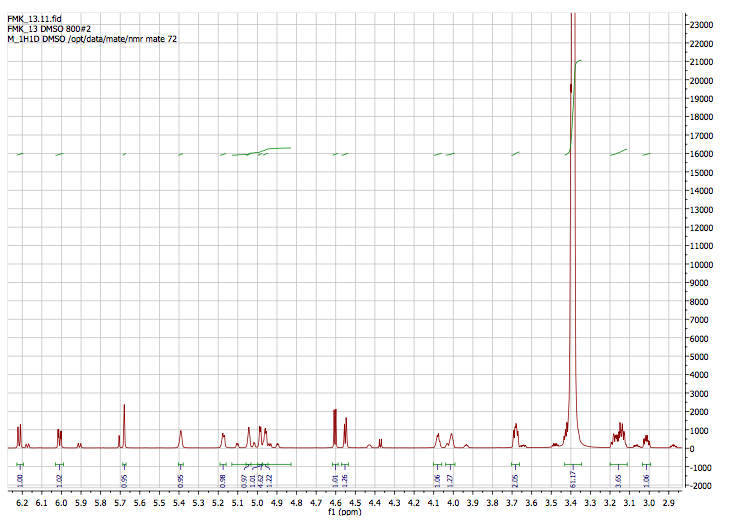


^13^CNMR CMP-8


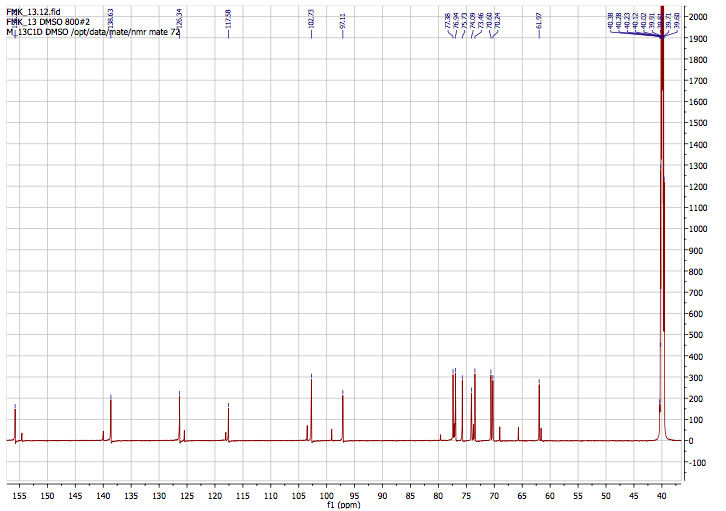


HSQC CMP-8


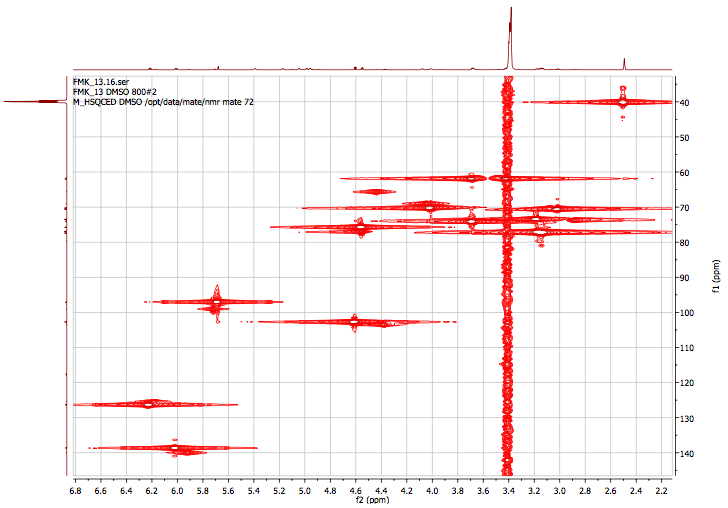


HMBC CMP-8


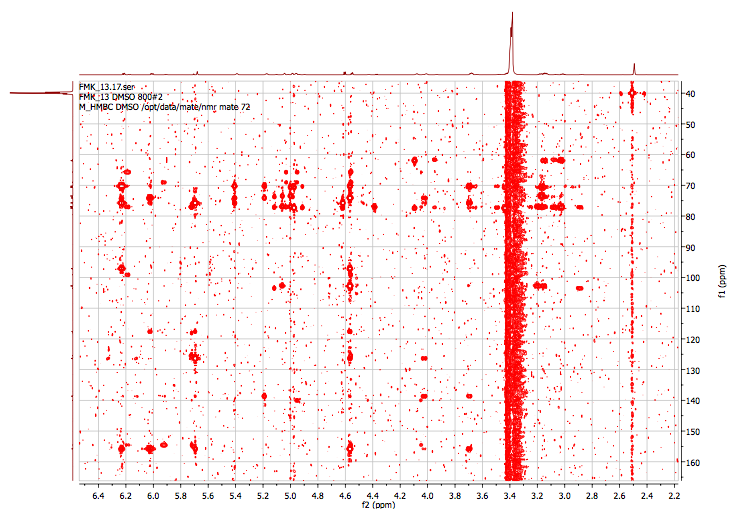


HRMS CMP-8
